# Intelligence

**Published:** 1836-07-01

**Authors:** 


### Intelligence.

### BIRMINGHAM ROYAL SCHOOL OF MEDICINE AND SURGERY.

AT a Quarterly Board of the Governors of this Institution, held June 1st, it was resolved, as a mark of respect to the President, to adjourn the Annual Meeting until the 26th of September—his eightieth birth-day. The following arrangements, in reference to the Prize Essays to be contended for by the Students, were determined upon. The Essays "*On the Influence of Air and Soil as affecting Health,*" premium Ten Guineas, offered by the Rev. Dr. Arnold. "*On Injuries of the Head,*" premium Five Guineas, offered by Mr. Meredith. "*Clinical Reports,*" premium Five Guineas, offered by Dr. Booth. To be sent in on or before September 1st next.

The Essays "*On the Influence upon Health of Alcoholic Drinks as an Article of Diet, including the Considerations whether any Quantity of any Kind be necessary for the Maintenance of Health even in the Case of those who are engaged in Laborious Occupations,*" premium Ten Guineas, offered by the Rev. J. A. James. To be sent in on or before January, 1837. To be awarded by the Rev. Chancellor Law, Rev. Dr. Jenner, Rev. Mr. Lawson, Rev. J. A. James, Dr. Booth, and Mr. S. Cox.

The Essay "*On the Capillary Vessels, viz. their Division, Conformation, Situation, Modes of Commuication, Structure, Physical and Vital Properties, Functions, and Morbid Anatomy.*" Premium Ten Guineas, offered by James Upfill, Esq. To be sent in on or before September, 1837.

WILLIAM SANDS COX, FRS. *Hon. Secretary.*

